# Gene expression profile for different susceptibilities to sound stimulation: a comparative study on brainstems between two inbred laboratory mouse strains

**DOI:** 10.1186/s12864-022-09016-3

**Published:** 2022-11-30

**Authors:** Lina Zhu, Deng Chen, Xin Lin, Ling Liu

**Affiliations:** Department of Neurology, West China Hospital, Sichuan University, Wai Nan Guo Xue Lane 37 #, Chengdu, 610041 Sichuan China

**Keywords:** Long noncoding RNAs, AGSz, S-IRA, SUDEP model

## Abstract

**Background:**

DBA/1 mice have a higher susceptibility to generalized audiogenic seizures (AGSz) and seizure-induced respiratory arrest (S-IRA) than C57/BL6 mice. The gene expression profile might be potentially related to this difference. This study aimed to investigate the susceptibility difference in AGSz and S-IRA between DBA/1 and C57BL/6 mice by profiling long noncoding RNAs (lncRNAs) and mRNA expression.

**Methods:**

We compared lncRNAs and mRNAs from the brainstem of the two strains with Arraystar Mouse lncRNA Microarray V3.0 (Arraystar, Rockville, MD). Gene Ontology (GO) and pathway analyses were performed to determine the potentially related biological functions and pathways based on differentially expressed mRNAs. qRT–PCR was carried out to validate the results.

**Results:**

A total of 897 lncRNAs and 438 mRNAs were differentially expressed (fold change ≥2, *P* < 0.05), of which 192 lncRNAs were upregulated and 705 lncRNAs were downregulated. A total of 138 mRNAs were upregulated, and 300 mRNAs were downregulated. In terms of specific mRNAs, Htr5b, Gabra2, Hspa1b and Gfra1 may be related to AGSz or S-IRA. Additionally, lncRNA Neat1 may participate in the difference in susceptibility. GO and pathway analyses suggested that TGF-β signaling, metabolic process and MHC protein complex could be involved in these differences. Coexpression analysis identified 9 differentially expressed antisense lncRNAs and 115 long intergenic noncoding RNAs (lincRNAs), and 2010012P19Rik and its adjacent RNA Tnfsf12-Tnfsf13 may have participated in S-IRA by regulating sympathetic neuron function. The results of the qRT–PCR of five selected lncRNAs (AK038711, Gm11762, 1500004A13Rik, AA388235 and Neat1) and four selected mRNAs (Hspa1b, Htr5b, Gabra2 and Gfra1) were consistent with those obtained by microarray.

**Conclusion:**

We concluded that TGF-β signaling and metabolic process may contribute to the differential sensitivity to AGSz and S-IRA. Among mRNAs, Htr5b, Gabra2, Hspa1b and Gfra1 could potentially influence the susceptibility. LncRNA Neat1 and 2010012P19Rik may also contribute to the different response to sound stimulation. Further studies should be carried out to explore the underlying functions and mechanisms of differentially expressed RNAs.

**Supplementary Information:**

The online version contains supplementary material available at 10.1186/s12864-022-09016-3.

## Introduction

C57BL/6 and DBA/1 mice are two different inbred strains that show diverse behavioral characteristics [1–4]. Specifically, DBA/1 mice have significantly higher susceptibility to audiogenic generalized seizures (AGSz), followed by seizure-induced respiratory arrest(S-IRA) [5]. This distinct feature may mimic the clinically observed sudden unexpected death in epilepsy (SUDEP) and thus makes DBA/1 mice relevant SUDEP models [6]. The underlying molecular mechanism of S-IRA following AGSz and S-IRA has not yet been clearly illustrated; however, many studies have provided valuable insights into these outcomes.

AGSz and S-IRA can be observed in many mouse strains and have been confirmed to indicate a unique form of seizure that originates in the brainstem. The physiological network in AGSz and S-IRA is thought to be common to different strains of mice, but the susceptibility to such seizures differs and may be influenced by many factors [7]. Considering only genetic background, the DBA mouse family (including DBA/1 and DBA/2 mice) show much higher susceptibility to AGSz than C57BL/6 mice, and several mutations have been found to be related to AGSz [5, 7]. However, genetic mutations cannot wholly explain the differences in susceptibility. Recent findings suggest that the serotonin (5-hydroxytryptamine, 5-HT) system plays an important role in AGSz and S-IRA. Selective serotonin reuptake inhibitors (SSRIs) were found to reduce S-IRA [8–10]. Western blotting also indicated that the tryptophan hydroxylase-2 (TPH 2) level in the DBA/1 mouse brainstem was significantly lower than that in the brainstem of the C57BL/6 J mouse [11]. Another report described that optogenetic activation of 5-HT neurons on the dorsal raphe of the brainstem reduced S-IAR in DBA/1 mice [12].

Other factors, such as time after birth, also influence susceptibility. Naturally, the AGSz and S-IRA of DBA/1 mice only exist in the first 5 weeks of age. However, they can be induced by repeated audio stimulation in early life, which was termed ‘priming’ [6]. Priming endows DBA/1 mice with much greater susceptibility, which may be reestablished in later life via the same stimulation [9]. These studies have suggested that brain development and the environment participate in susceptibility, and thus, epigenetic regulation should be considered.

However, direct evidence for epigenetic regulation is limited. In this study, we aimed to compare the differences in mRNA and long noncoding RNA (lncRNA) expression profiles in the brainstem of DBA/1 and C57BL/6 mice, which may provide insights into the potential epigenetic regulation that mediates AGSz and S-IRA.

## Materials and methods

**Fig. 1:**
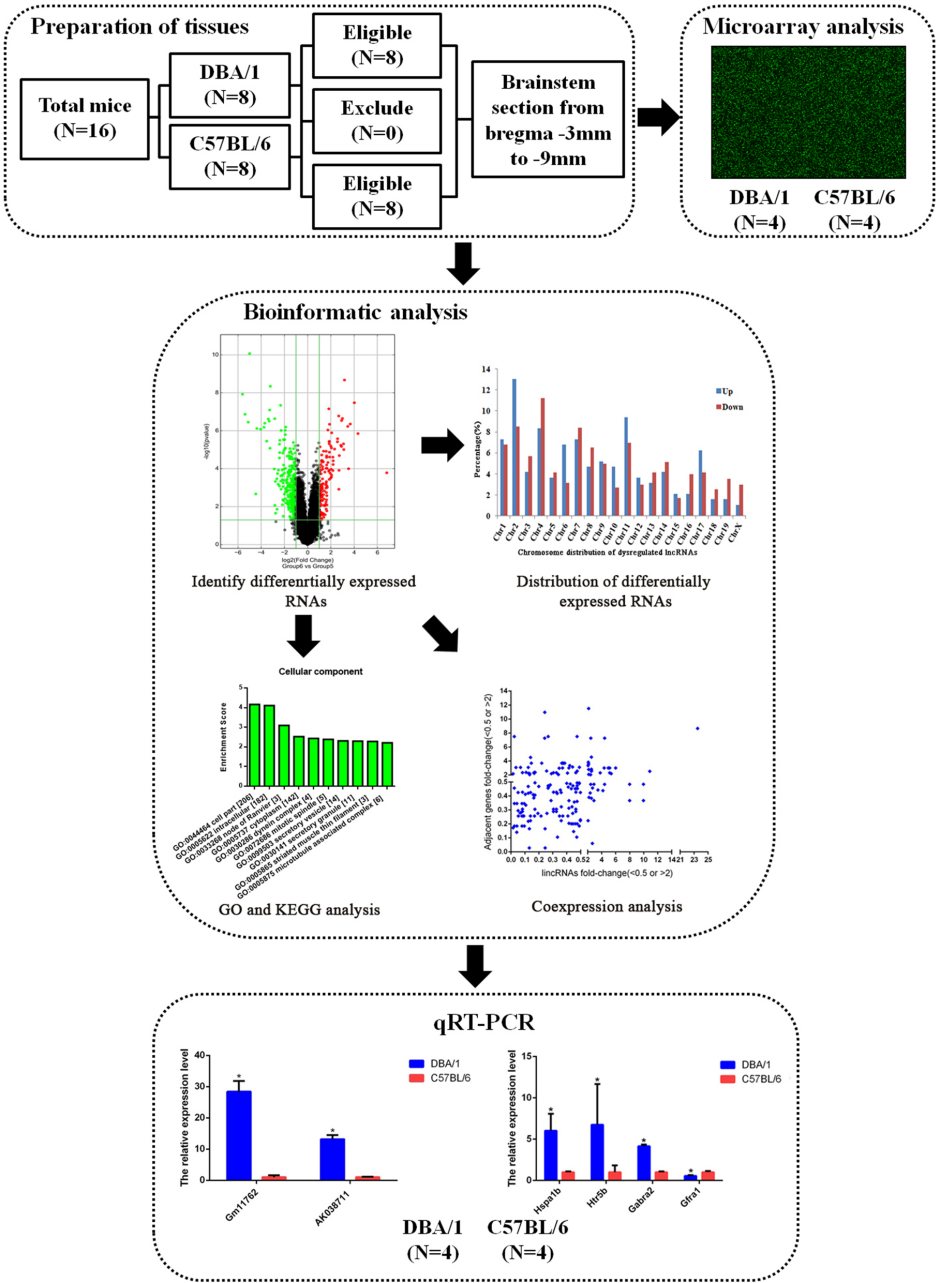
The flowchart of experimental procedure. The study contained four procedural modules, including preparation of tissues, microarray analysis, bioinformatics analysis, and qRT-PCR

In this study, we established four procedural modules, including modules for the preparation of tissues, a microarray analysis, a bioinformatics analysis, and qRT–PCR (Fig. 1).

### Preparation of tissues

All experimental operations and procedures with animals were performed in accordance with the Guidelines of Animal Care and Use Committee of Sichuan University West China Hospital. The study was not preregistered. Since the AGSz response without priming in DBA/1 mice was quite stable on different postnatal days (PNDs) from 21 to 112 PNDs [13], we purchased PND 28-30 DBA/1JNCrlj and C57BL/6JNifdc male mice from the Charles River Laboratories Experimental Animal Center (Beijing, China). The DBA/1 and C57BL/6 mice were housed in standard laboratory cages (4 mice per cage), and the mice had free access to water and food in a temperature-controlled room (21 °C-25 °C). All animals were maintained under a 12-h light/dark cycle. We evaluated only male DBA/1 mice because 1. previous reports indicated that they are slightly more susceptible to AGSz and S-IRA than females [9] and 2. the susceptibility to seizure may be influenced by ovarian hormones in female mice [14]. Since AGSz and audio stimulation potentially affect RNA expression, these mice were housed in specific pathogen-free (SPF) conditions for 1 week without testing for AGSz. The DBA/1 (*n* = 8) and C57BL/6 (n = 8) mice were decapitated at the age of 5 weeks under anesthesia with isoflurane inhalation, and the whole brainstem between bregma − 3 mm and bregma − 9 mm was taken as described in a previous study [11]. The brainstem samples were immediately frozen in liquid nitrogen and then stored at − 80 °C for later use. Four samples from each strain were used for the microarray analysis, and the other 4 samples were used for verification by qRT–PCR.

### RNA extraction

Total RNA was extracted from DBA/1 and C57BL/6 brainstem tissues using TRIzol reagent (Invitrogen Life Technologies, Carlsbad, CA). RNA quantification and quality were evaluated with a NanoDrop ND-1000 spectrometer (Thermo Fisher Scientific, USA). Standard denaturing agarose gel electrophoresis was applied for the measurement of RNA integrity.

### Microarray analysis

An Arraystar mouse lncRNA Microarray V3.0 (Arraystar, Rockville, MD) was used in our study, which could detect about 35,923 lncRNAs and 24,881 coding transcripts. The microarray analysis was performed by KangChen Biotech (Shanghai, China). The lncRNAs were annotated by using authoritative public transcriptome databases (NCBI Refseq 2014, UCSC Known Gene 6.0, Ensembl 38.71 and Genbank) and landmark publications (see Additional file 1), while coding mRNAs were collected from Collaborative Consensus Coding Sequence (CCDS) Project. Sample labeling and array hybridization were carried out on the basis of the Agilent one-color microarray-based gene expression analysis experimental scheme (Agilent Technology). First, we extracted rRNA from total RNA to obtain purified mRNA (RNA-ONLY Eukaryotic RNA Isolation Kit, Epicentre). Second, we used a random primer method to amplify and transcribe each sample into fluorescent cRNA (Arraystar flash RNA labeling kit, Arraystar). Following this, an RNeasy Mini Kit (Qiagen) was utilized to purify the labeled cRNA, the concentration and activity of which were further determined with a NanoDrop ND-1000. Then, the labeled cRNA was hybridized onto microarray slides, and the hybridized arrays were washed, fixed and scanned with an Agilent DNA Microarray Scanner (part number G2505C).

### Bioinformatic analysis

The Agilent Feature Extraction software (version 11.0.1.1) and the GeneSpring GX v12.1 software package (Agilent Technologies) were applied to analyze array images and quantile normalization of the raw data, respectively. Quantile normalization was performed as follows: The expression values of specific RNAs were listed in a matrix where each row represents one RNA and each column represents one sample. For each sample, the original values of different RNAs were sorted in ascending order in the column. The mean of the sorted order across each row was obtained, and then, the value of each row was replaced by this mean. Finally, the modified matrix in the previous step was rearranged to follow the same order as the input matrix.

A hierarchical clustering map and volcano plot were created to present the profiles of differentially expressed lncRNAs and mRNAs. Gene Ontology (GO) analysis and Kyoto Encyclopedia of Genes and Genomes (KEGG) analysis were performed to identify the potential biological functions and pathways in which the differentially expressed mRNAs were enriched.

We performed coexpression analysis to show the aberrantly expressed antisense lncRNAs with their sense mRNAs and the different expression of long noncoding intergenic RNA (lincRNA) with their nearby coding genes, which was considered important in a bioinformatics analysis [15]. We first subdivided lncRNAs into 6 subgroups, which were defined as follows:

1. Sense overlapping, the lncRNA exon overlapped a coding transcript exon in the same genomic strand;
2. Intronic, the lncRNA overlapped an intron of a coding transcript in the same genomic strand;
3. Natural antisense, the lncRNA was transcribed from the antisense strand and overlapped a coding transcript;
4. Nonoverlapping antisense, the lncRNA was transcribed from the antisense strand without overlapping an exon;
5. Bidirectional, the lncRNA was oriented head-to-head with a coding transcript within 1000 bp;
6. Intergenic, there were no overlapping or bidirectional coding transcripts near the lncRNA.

In the second part of the coexpression analysis, we included all intergenic lncRNAs and set the distance from the lncRNAs to nearby genes to be < 300 kb to identify lincRNAs. As a major lncRNA subtype, a lincRNA is transcribed from intergenic regions and is involved in regulating the expression of adjacent genes [16].

### Quantitative real-time PCR

Quantitative real-time polymerase chain reaction (qRT–PCR) were performed to verify different expression patterns of lncRNAs and mRNAs obtained in the microarray analysis by a SYBR green PCR kit and a ViiA 7 Real-time PCR System (Applied Biosystems). All the expression levels of lncRNAs and mRNAs were normalized to the level of the internal reference gene (GAPDH). In this study, dysregulated lncRNAs used for verification were selected primarily on the basis of fold change. We also considered homology between mice and humans and selected some lncRNAs of interest in our study. For the selection of mRNAs, we took the results of GO and KEGG analyses into account and then combined these results with potential known mechanisms that may contribute to differences in AGSz or S-IRA. The sequences of the primers are listed in Table 1.

**Table 1:** The sequences of lncRNA and mRNA primers used in the study

Differential expression	Gene name	Primers (5′-3′) (F=Forward; R = Reverse)	Amplicon size (bp)
lncRNAs	uc007uzp.1	F: CACCAAATGGGCTGGACAA	197
		R: GGCTAAAGGCAGACTGGAATC	
	NR_045099	F: GCCATCCAGTTCCATCTTTCT	143
		R: GCCCCTGTCTGTTCTCCATAA	
	NR_015498	F: CAACGGAGTTACTATGGGTCG	279
		R: GAGGCTACGGGTGAGGTTAT	
	NR_033305	F: CAAGGAACTTTGGTCGTAGC	86
		R: AGCAATACAACAATGACTAAGACA	
	NR_003513	F: GGTTGTTTTGTGAGTGTGCTTA	169
		R: GGGGAGGAAAATGGTTAGTG	
mRNAs	Hspa1b	F: TATAGTCTAGCTGCCCAGTTCC	75
		R: CAGTGCCAAGACGTTTGTTT	
	Htr5b	F: GGTGGTGCTCTTCGTCTACT	179
		R: AGTCTCCGCTTGTCTGGAAG	
	Gabra2	F: ACAGTCCAAGCCGAATGTCC	138
		R: AACGGAGTCAGAAGCATTGTAAGT	
	Gfra1	F: CCACTCCTGGATTTGCTGAT	152
		R: CTGAAGTTGGTTTCCTTGCC	
Internal control	GAPDH	F: CACTGAGCAAGAGAGGCCCTAT	144
		R: GCAGCGAACTTTATTGATGGTATT	

### Statistical analyses

Differentially expressed lncRNAs and mRNAs were identified as those with a fold change ≥2.0 and a *P* value < 0.05. We performed false discovery rate (FDR) correction to minimize false-positives. Another analysis based on a fold change ≥2.0 and an FDR < 0.05 was performed as a sensitivity test. The qRT–PCR results are shown as the relative expression levels. We used an independent set of 4 DBA/1 and 4 C57BL/6 mice for qRT–PCR to verify the microarray findings. By setting the expression value of the target genes in the C57BL/6 control group to 1, the expression level in the DBA/1 mouse group is reported the fold change compared with the control group.

## Results

### Differentially expressed lncRNAs and mRNAs in DBA/1 mice compared with C57BL/6 mice

**Fig. 2:**
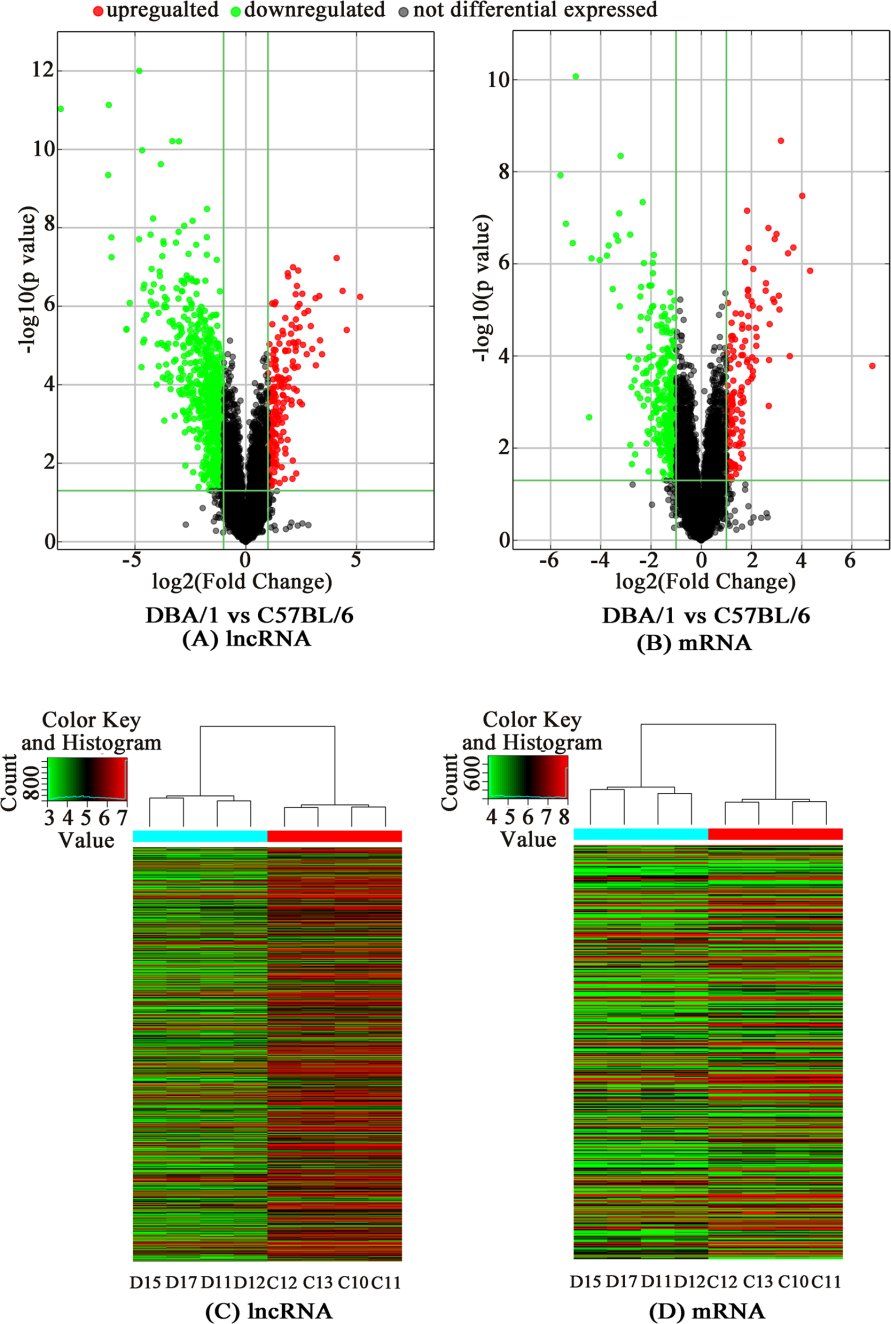
Differentially expressed lncRNAs and mRNAs in DBA/1 mice compared with C57BL/6 mice. The Volcano Plots of lncRNA (**a**) and mRNA (**b**) expression; Hierarchical clustering of differentially expressed lncRNA (**c**) and mRNA (**d**). ‘red’ indicates high relative expression, and ‘green’ indicates low relative expression. ‘C’ and ‘D’ respectively represent C57BL/6 and DBA/1 group (each group with four mice)

The microarray analysis led to the identification of a total of 897 significantly differentially expressed lncRNAs (192 up- and 705 downregulated) and 438 differentially expressed mRNAs (138 up- and 300 downregulated). The details of all these differentially expressed RNAs are showed in the supplementary material (Additional files 1 and 2). The 20 most differentially expressed lncRNAs and mRNAs between DBA/1 and C57BL/6 mice are listed in Table 2 and Table 3, respectively. Figure 2 shows the volcano plots and hierarchical clustering analysis depicting the expression levels of the distinguishable lncRNAs and mRNAs.

**Table 2:** Top 20 differentially expressed lncRNAs between DBA/1 and C57BL/6 mice

Up-regulated lncRNAs				Down-regulated lncRNAs			
Seqname	GeneSymbol	Fold Change^a^	FDR	Seqname	GeneSymbol	Fold Change^a^	FDR
ENSMUST00000117627	Gm14201	35.75	0.0002	uc009bwo.2	AK005187	326.12	0.0000
uc007uzp.1	AK038711	23.50	0.0005	uc008pwq.2	1500004A13Rik	73.85	0.0000
NR_045099	Gm11762	20.65	0.0001	NR_015498	1500004A13Rik	72.49	0.0000
ENSMUST00000173149	H2-Bl	17.14	0.0000	NR_033305	AA388235	66.92	0.0000
ENSMUST00000174778	Gm10499	10.86	0.0013	AK047380	AK047380	66.64	0.0000
uc008ryr.1	AK019984	10.21	0.0008	AK047372	AK047372	41.87	0.0005
ENSMUST00000137728	AI847159	9.97	0.0002	AK143879	AK143879	41.70	0.0005
uc008mqp.1	AK085768	8.95	0.0021	AK053631	AK053631	37.72	0.0002
ENSMUST00000161336	Agl	8.87	0.0002	TCONS_00025043	XLOC_018501	28.09	0.0000
ENSMUST00000151051	Gm14029	7.99	0.0010	AK043180	AK043180	27.94	0.0000
ENSMUST00000180930	Gm26793	7.63	0.0005	NR_045175	Smc2os	26.11	0.0022
ENSMUST00000142000	Ift140	7.03	0.0003	uc008pws.2	1500004A13Rik	25.55	0.0000
ENSMUST00000129337	Gm11508	6.73	0.0013	AK040275	AK040275	24.70	0.0008
ENSMUST00000174018	Grm7	6.25	0.0003	AK053990	AK053990	24.55	0.0001
ENSMUST00000176545	AA465934	5.99	0.0009	uc008ouc.1	AK007174	24.15	0.0004
AK155705	AK155705	5.85	0.0001	AK157804	AK157804	23.90	0.0008
AK017289	AK017289	5.79	0.0099	ENSMUST00000178906	Gm10593	23.88	0.0001
ENSMUST00000181014	D330041H03Rik	5.40	0.0091	AK047207	AK047207	22.35	0.0003
AK136371	AK136371	5.39	0.0005	AK037460	AK037460	19.67	0.0000
AK084340	AK084340	5.37	0.0002	AK157092	AK157092	19.58	0.0002

Notes: lncRNAs, long non-coding RNAs; FDR, false discovery rate. ^a^ DBA/1 mice vs. C57BL/6 mice

**Table 3:** Top 20 differentially expressed mRNAs between DBA/1 and C57BL/6 mice

Up-regulated mRNAs				Down-regulated mRNAs			
Seqname	GeneSymbol	Fold Change^a^	FDR	Seqname	GeneSymbol	Fold Change^a^	FDR
NM_001037713	Xaf1	112.48	0.0159	NM_025617	Tceanc2	48.72	0.0000
NM_001163810	Tescl	20.19	0.0008	NM_010500	Ier5	41.87	0.0002
NM_001142938	AK010878	16.23	0.0001	NM_001161411	Trappc12	34.68	0.0004
NM_011414	Slpi	12.71	0.0004	NM_024472	Gltpd1	31.90	0.0000
NM_009247	Serpinale	11.51	0.0123	NM_001033149	Ttc9	22.04	0.0652
NM_001083918	Gm13139	10.97	0.0005	NM_001039533	Pdxdc1	20.65	0.0005
NM_001111119	Ccnb1ip1	9.03	0.0000	NM_001145899	Slc15a2	16. 47	0.0006
NM_001001490	Oxgr1	8.66	0.0027	NM_198619	Zfp933	13.51	0.0005
NM_053127	Pcdhb2	8.51	0.0018	NM_207533	Dbx2	12.88	0.0004
NM_019788	Bloc1s6	7.98	0.0003	NM_011562	Tdgf1	11.52	0.0015
NM_022420	Gprc5b	7.59	0.0003	NM_032002	Nrg4	10.46	0.0003
NM_029865	Ocel1	7.51	0.0022	NM_183167	AI987944	9.91	0.0003
NM_001103158	Gm13242	7.27	0.0021	NM_015800	Crim1	9.62	0.0002
NM_026645	Iqcf3	6.61	0.0044	NM_145594	Fgl1	9.47	0.0024
NM_009244	Serpina1b	6.50	0.0132	NM_001130176	Tnnt2	9.25	0.0000
NM_175296	Mael	6.44	0.0469	NM_025922	Itpa	7.34	0.0124
NM_175537	Zbtb38	6.38	0.0003	NM_030707	Fcrls	7.11	0.0003
NM_153568	Lrrc66	5.97	0.0014	NM_019440	Irgm2	7.08	0.1372
NM_010478	Hspa1b	5.92	0.0016	NM_011723	Xdh	6.85	0.0292
NM_001127188	Zfp534	4.99	0.0026	NM_001143686	Apol11b	6.77	0.2280

Notes: FDR, false discovery rate. ^a^ DBA/1 mice vs. C57BL/6 mice

**Fig. 3:**
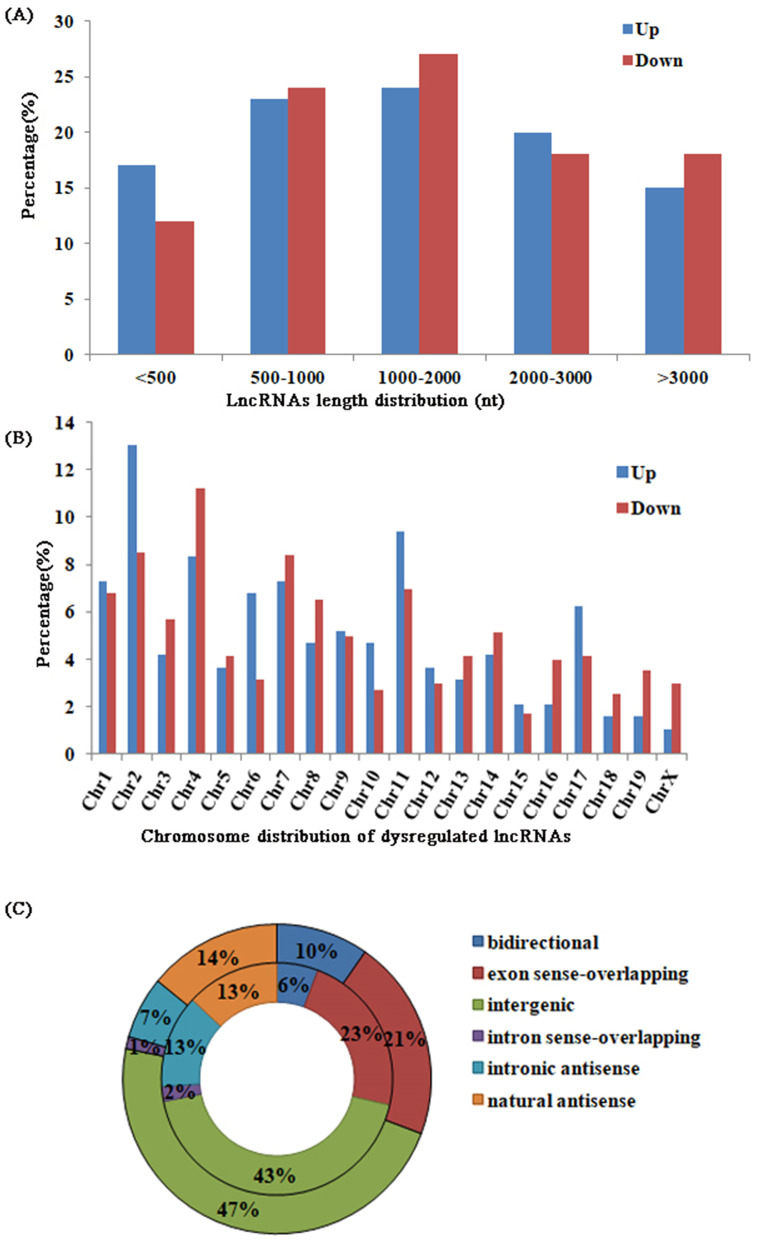
**a** The percentage of the length distribution of differentially expressed lncRNAs; (**b**) The percentage of the chromosome distribution of differentially expressed lncRNAs; (**c**) The types of differentially expressed lncRNAs. Upregulated lncRNAs were showed in the inner circle, and downregulated lncRNAs were described in the outer circle

The detailed characteristics of the lncRNAs are also described (Fig. 3): the length distribution of the up- and downregulated lncRNAs was greatest in the 1000-2000 nt bin (24 and 27%, respectively); the most frequent transcriptional locations of up- and downregulated lncRNAs were chromosomes 2 and 4 (13 and 11%, respectively); and 43% of up- and 47% of downregulated lncRNAs were intergenic.

The results of the sensitivity test based on an FDR < 0.05 are available in the supplementary material.

### GO and pathway analysis of differentially expressed mRNAs

**Fig. 4:**
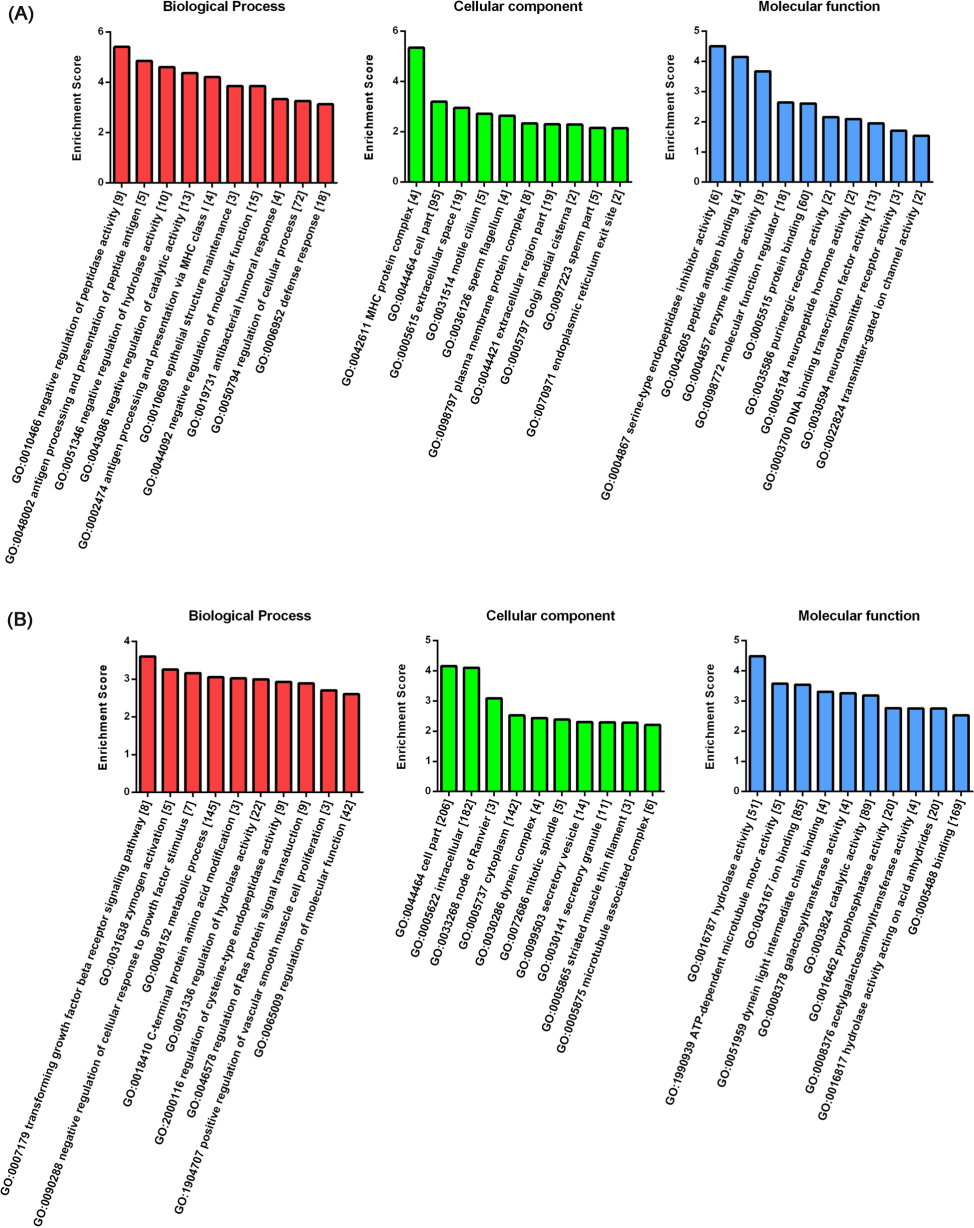
GO analysis comparing DBA/1 group with C57BL/6 group. **a** Top 10 enriched GO terms from upregulated mRNAs in biological process, cellular component, and molecular function. **b** Top 10 enriched GO terms from downregulated mRNAs in biological process, cellular component, and molecular function

GO analysis was performed to assess the biological functions of genes and gene products, which were classified into biological processes (BP), cellular components (CC), and molecular function (MF). The most highly enriched GO terms targeted by upregulated genes were negative regulation of peptidase activity (GO: 0010466) in BP, MHC protein complex (GO: 0042611) in CC and serine-type endopeptidase inhibitor activity (GO:0004867) in MF (Fig. 4a). The most enriched GO terms among downregulated genes were transforming growth factor beta receptor signaling pathway (GO: 0007179) in BP, cell part (GO:0044464) in CC, and hydrolase activity (GO: 0016787) in MF (Fig. 4b).

KEGG was performed to identify genes involved in different biological pathways [17–19]. This analysis revealed 28 pathways enriched with significantly differentially expressed genes, including 20 pathways corresponding to upregulated transcripts and 8 corresponding to downregulated transcripts. The most correlated pathway among upregulated genes was Type I diabetes mellitus, and among downregulated genes, it was natural killer cell-mediated cytotoxicity. The details are shown in Table 4.

**Table 4:** Pathways identified from comparison between DBA/1 mice and C57BL/6 mice

lncRNA types	KEGG pathways	Associated genes
Up-regulated lncRNAs	1. Type I diabetes mellitus	*H2-D1,H2-DMA,H2-Q6,H2-T24,LTA*
	2. Viral myocarditis	*CXADR,H2-D1,H2-DMA,H2-Q6,H2-T24*
	3. Herpes simplex virus 1 infection	*H2-D1,H2-DMA,H2-Q6,H2-T24,LTA,ZFP457,ZFP51,ZFP658,ZFP961,ZFP963*
	4. Antigen processing and presentation	*H2-D1,H2-DMA,H2-Q6,H2-T24,HSPA1B*
	5. Cell adhesion molecules	*CLDN13,H2-D1,H2-DMA,H2-Q6,H2-T24,MPZL1*
	6. Allograft rejection	*H2-D1,H2-DMA,H2-Q6,H2-T24*
	7. Graft-versus-host disease	*H2-D1,H2-DMA,H2-Q6,H2-T24*
	8. Autoimmune thyroid disease	*H2-D1,H2-DMA,H2-Q6,H2-T24*
	9. Neuroactive ligand-receptor interaction	*CORT,GABRA2,GRIN3A,HTR5B,PTAFR,PTGFR,PYY*
	10. *Staphylococcus aureus* infection	*DEFA17,DEFA24,H2-DMA,PTAFR*
	11. Kaposi sarcoma-associated herpesvirus infection	*ANGPT2,GM5741,H2-D1,H2-Q6,H2-T24*
	12. Human T-cell leukemia virus 1 infection	*H2-D1,H2-DMA,H2-Q6,H2-T24,LTA*
	13. Endocytosis	*H2-D1,H2-Q6,H2-T24,HSPA1B,IQSEC1*
	14. Phagosome	*H2-D1,H2-DMA,H2-Q6,H2-T24*
	15. Nicotine addiction	*GABRA2,GRIN3A*
	16. Calcium signaling pathway	*CAMK4,HTR5B,PTAFR,PTGFR*
	17. Serotonergic synapse	*GM5741,HTR5B,TPH2*
	18. Epstein-Barr virus infection	*H2-D1,H2-DMA,H2-Q6,H2-T24*
	19. Human immunodeficiency virus 1 infection	*GM5741,H2-D1,H2-Q6,H2-T24*
	20. Glycerolipid metabolism	*DGKB,LPL*
Down-regulated lncRNAs	1. Natural killer cell mediated cytotoxicity	*GZMB,KLRA7,NFATC2,PPP3R2,SOS1,TNFSF10*
	2. Endocrine and other factor-regulated calcium reabsorption	*CLTA,KLK1B3,KLK1B8,KLK1B9*
	3. Starch and sucrose metabolism	*GBE1,GPI1,SIS*
	4. Renin-angiotensin system	*KLK1B3,KLK1B8,KLK1B9*
	5. Glycosphingolipid biosynthesis	*KLK1B3,KLK1B8,KLK1B9*
	6. Cell cycle	*CHEK2,CUL1,MCM6,ORC6,PTTG1*
	7. Amyotrophic lateral sclerosis (ALS)	*MAP3K5,PPP3R2,TNFRSF1B*
	8. T cell receptor signaling pathway	*4930544G11RIK,NFATC2,PPP3R2,SOS1*

The sensitivity test for GO and KEGG was showed in the supplemental files based on a fold change ≥2.0 and an FDR < 0.05.

### Coexpression analysis

In this study, nine differentially expressed antisense lncRNAs were found between the DBA/1 group and the C57BL/6 control group. Among these lncRNAs, 2010012P19Rik was the most downregulated lncRNA (fold change = 6.09, FDR < 0.001) with its nearby gene, *Tnfsf12-Tnfsf13* (fold change =2.04, FDR = 0.025). Other differentially expressed antisense lncRNAs and their paired sense mRNAs are shown in Table 5.

**Table 5:** Differentially expressed antisense lncRNAs and nearby coding gene

Seqname of lncRNA	Gene symbol	Fold change^a^ (lncRNAs)	Regulation of lncRNA	Genome relationship	Nearby gene seqname	Nearby gene Symbol	Fold change^a^ (mRNAs)	Regulation of mRNA
ENSMUST00000145435	2010012P19Rik	6.0917526	down	natural antisense	NM_001034097	*Tnfsf12-Tnfsf13*	2.0415635	down
AK017289	AK017289	5.792853	up	natural antisense	NM_001267808	*H2-L*	2.8392743	up
AK017289	AK017289	5.792853	up	natural antisense	NM_010380	*H2-D1*	2.6094529	up
AK155933	AK155933	4.4353006	down	intronic antisense	NM_146191	*Lrrk1*	2.53359	down
AK087052	AK087052	3.4286643	up	intronic antisense	NM_001035242	*Trpm3*	2.0411979	down
AK087052	AK087052	3.4286643	up	intronic antisense	NM_001035243	*Trpm3*	2.0100955	down
AK149710	AK149710	3.163844	down	natural antisense	NM_008850	*Pitpna*	2.4984672	down
AK158573	AK158573	2.5693914	down	natural antisense	NM_028803	*Gbe1*	2.5839838	down
ENSMUST00000148180	Gm15396	2.5550359	down	natural antisense	NM_008437	*Napsa*	4.8828493	down
AK007047	AK007047	2.510748	down	natural antisense	NM_001200023	*Zfp963*	3.3589015	up
ENSMUST00000124513	Gm15247	2.064782	down	natural antisense	NM_183151	*Mid1*	3.2269554	down

Notes: lncRNAs, long non-coding RNAs. ^a^ DBA/1 mice vs. C57BL/6 mice

**Fig. 5:**
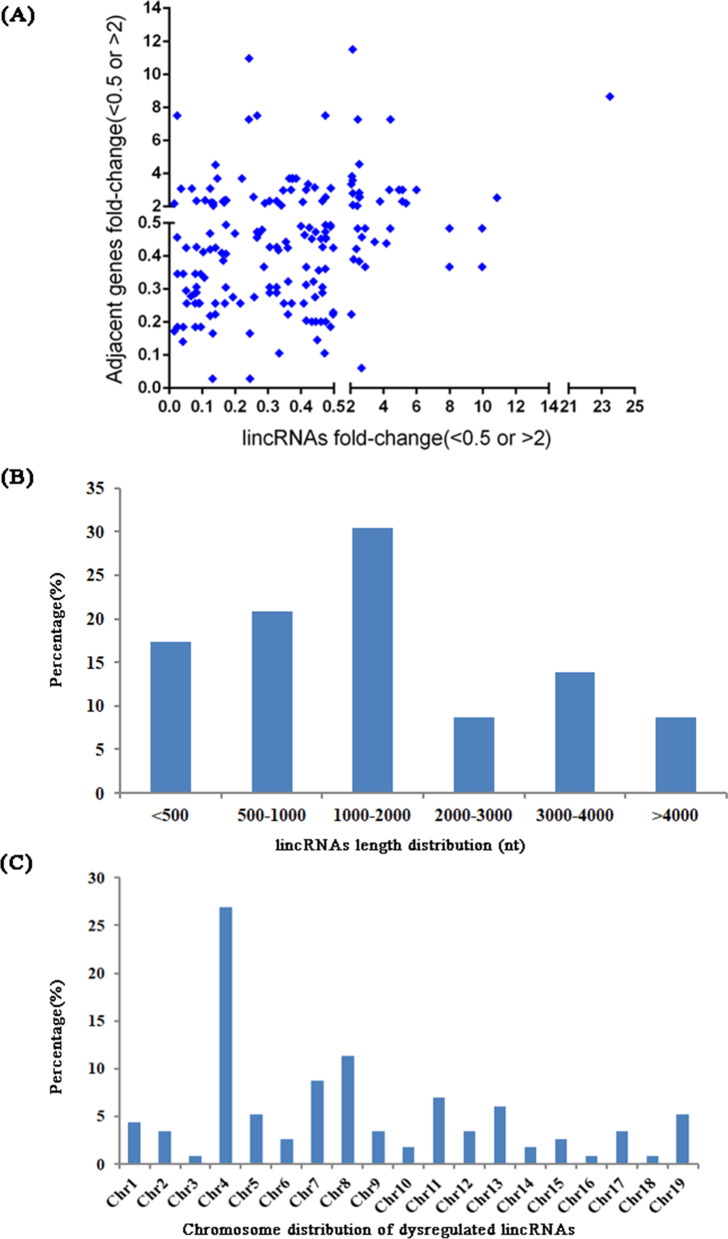
**a** Fold change of significantly dysregulated lincRNA and their differentially expressed adjacent mRNAs for DBA/1mice vs. C57BL/6 mice; (**b**) The percentage of the length distribution of differentially expressed lincRNAs; (**c**) The percentage of the chromosome distribution of differentially expressed lincRNAs

A total of 115 long intergenic noncoding RNAs (lincRNAs) were found to be differentially expressed. Among them, approximately 68.5% of the lincRNAs and their adjacent coding genes were changed in the same direction (which means that both lncRNAs and mRNAs were upregulated or that both were downregulated), including 12.9% upregulated and 55.6% downregulated pairs. Eight percent of dysregulated lincRNAs were upregulated and their paired mRNAs were downregulated, while 22.5% of lincRNAs were downregulated, with their nearby mRNAs upregulated. Fold-changes of differential expression among lincRNA-gene pairs are presented in a scatter plot (Fig. 5a). The length distribution of differentially expressed lincRNAs was greatest in the 1000-2000 nt bin (30.4%), and these differential lincRNAs were most frequently transcribed from chromosome 4 (27%). The details are shown in Fig. 5b and Fig. 5c, respectively. The 20 most differentially expressed lincRNAs and their adjacent mRNAs are presented in Table 6.

**Table 6:** Top 20 differentially expressed lincRNAs and adjacent mRNAs

Seqname of lncRNA	Gene symbol	Fold change^a^ (lncRNAs)	Regulation of lncRNA	Genome relationship	Nearby gene seqname	Nearby gene Symbol	Fold change^a^ (mRNAs)	Regulation of mRNA
NR_033305	AA388235	66.9199947	down	downstream	NM_010386	*H2-Dma*	2.170267	up
NR_033305	AA388235	66.9199947	down	upstream	NM_019420	*B3galt4*	5.7973092	down
AK047372	AK047372	41.8748727	down	downstream	NM_001163042	*Haus8*	2.1908769	down
AK047372	AK047372	41.8748727	down	downstream	NM_029865	*Ocel1*	7.5104853	up
AK143879	AK143879	41.7014068	down	upstream	NM_001193667	*Gm1987*	2.8931939	down
AK143879	AK143879	41.7014068	down	upstream	NM_001277167	*Gm12429*	5.3971264	down
TCONS_00025043	XLOC_018501	28.0948375	down	upstream	NM_008861	*Pkd2*	3.0839338	up
AK053990	AK053990	24.5521064	down	upstream	NM_030707	*Fcrls*	7.1055169	down
ENSMUST00000178906	Gm10593	23.884969	down	downstream	NM_001193667	*Gm1987*	2.8931939	down
ENSMUST00000178906	Gm10593	23.884969	down	downstream	NM_001277167	*Gm12429*	5.3971264	down
uc007uzp.1	AK038711	23.496569	up	downstream	NM_001001490	*Oxgr1*	8.6586855	up
AK037460	AK037460	19.6676915	down	upstream	NM_001008232	*Asap3*	2.3508325	down
AK037460	AK037460	19.6676915	down	upstream	NM_011542	*Tcea3*	3.3860996	down
uc029usn.1	Gm5859	19.0222774	down	upstream	NM_001085530	*Gm13298*	3.895603	down
AK037363	AK037363	15.2204905	down	downstream	NM_008911	*Ppox*	3.5903657	down
AK136314	AK136314	14.6596357	down	upstream	NM_008861	*Pkd2*	3.0839338	up
NR_040401	C920006O11Rik	13.1823016	down	downstream	NM_025274	*Dppa5a*	3.5029739	down
uc029urz.1	DQ551946	12.8034244	down	downstream	NM_001085530	*Gm13298*	3.895603	down
AK141495	AK141495	12.6885049	down	downstream	NM_001193667	*Gm1987*	2.8931939	down
AK141495	AK141495	12.6885049	down	downstream	NM_001277167	*Gm12429*	5.3971264	down
ENSMUST00000151374	Snhg3	12.1370958	down	downstream	NM_001081651	*Rab42*	3.2691229	down
ENSMUST00000151374	Snhg3	12.1370958	down	upstream	NM_001081211	*Ptafr*	2.3332466	up
ENSMUST00000151374	Snhg3	12.1370958	down	upstream	NM_001161797	*Phactr4*	3.4588717	down
ENSMUST00000151374	Snhg3	12.1370958	down	upstream	NM_026039	*Med18*	2.3377675	down
ENSMUST00000121728	Gm13301	11.4196096	down	upstream	NM_001085530	*Gm13298*	3.895603	down
ENSMUST00000178043	Gm3892	11.2523847	down	upstream	NM_001085530	*Gm13298*	3.895603	down
ENSMUST00000174778	Gm10499	10.8643014	up	downstream	NM_008207	*H2-T24*	2.5411856	up
ENSMUST00000107991	Gm3892	10.8301037	down	upstream	NM_001085530	*Gm13298*	3.895603	down
AK052053	AK052053	10.6618917	down	downstream	NM_001193667	*Gm1987*	2.8931939	down
AK052053	AK052053	10.6618917	down	downstream	NM_001277167	*Gm12429*	5.3971264	down

Notes: lincRNAs, long intergenic noncoding RNAs. ^a^ DBA/1 mice vs. C57BL/6 mice

A sensitivity test was also performed on the basis of an FDR < 0.05, and the results are also given in the supplementary material.

### Validation of differentially expressed lncRNAs and mRNAs

**Fig. 6:**
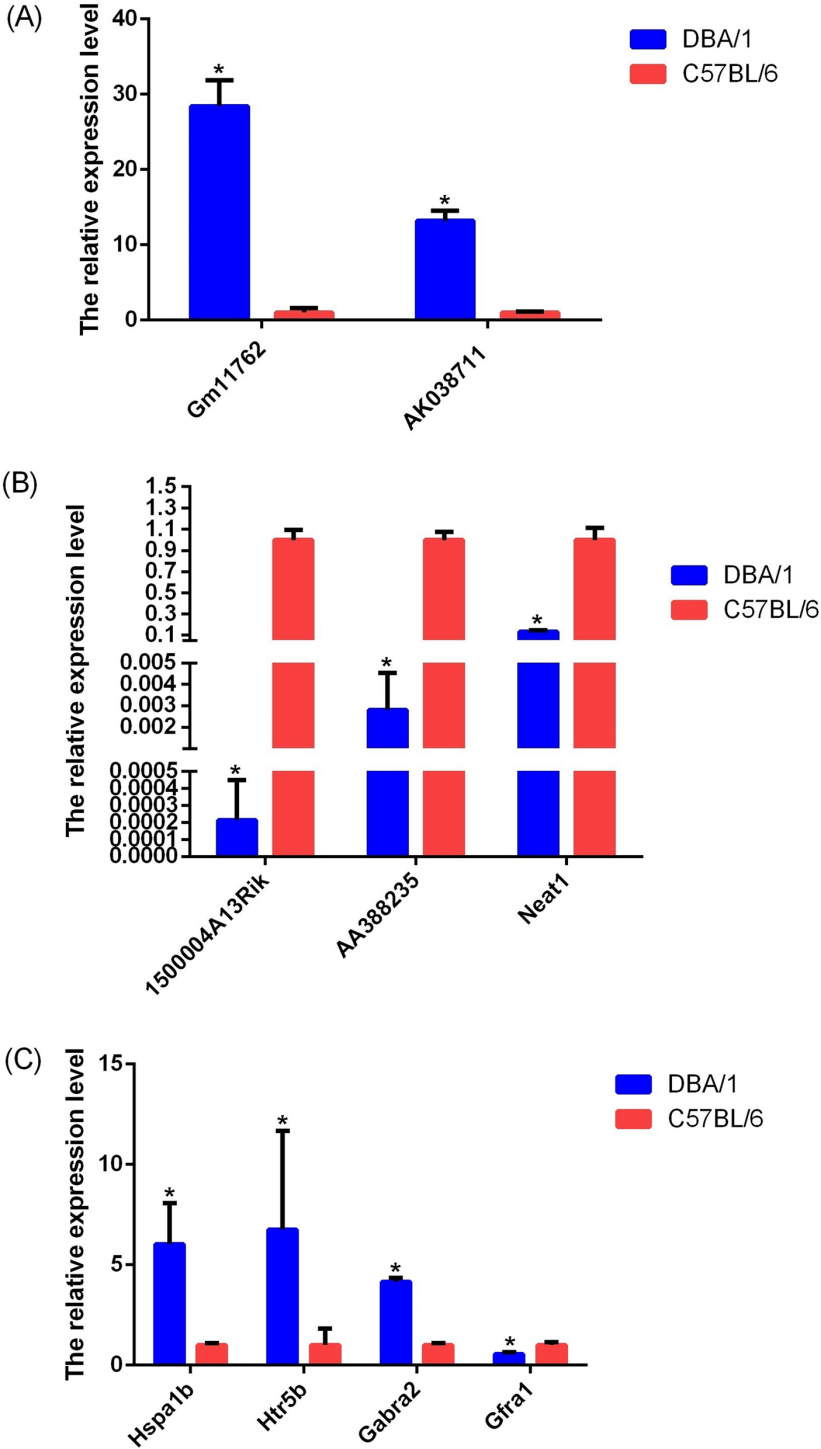
The qRT-PCR vadidation of differentially expressed lncRNAs and mRNAs between DBA/1 mice with C57BL/6. **a** The qRT-PCR results of up-regulated lncRNAs; (**b**) The qRT-PCR results of down-regulated lncRNAs; (**c**) The qRT-PCR results of differentially expressed mRNAs. By setting the expression value of target genes in C57BL/6 control group at 1, the expression level of which in DBA/1 mice group was the fold change relative to control group. Significant levels were indicated by * (*P* < 0.05). The results of qRT-PCR were consistent with that in microarray analysis (*n* = 4 animals/group)

Differentially expressed genes were selected to be analyzed by qRT–PCR, including five lncRNAs (two upregulated and three downregulated) and four mRNAs (three upregulated and one downregulated). According to the qRT–PCRs, the expression of AK038711 (uc007uzp.1) and Gm11762 (NR_045099) was upregulated (Fig. 6a), whereas that of 1500004A13Rik (NR_015498), AA388235 (NR_033305) and Neat1 (NR_003513) was downregulated (Fig. 6b) in DBA/1 mice compared with C57BL/6 mice. This result was consistent with the microarray assay.

Through a GO and KEGG analysis, we identified 4 potentially related transcripts for validation according to known mechanisms that are potentially related to AGSz or S-IRA. They were Hsp1a, Htr5b, Gabra2 and Gfra1. In our enrichment analysis, Hsp1a was found to be involved in multiple process of regulation on multiple enzyme activity (GO) and stress responses (GO), while Gfra1 was located on axon (GO) and exert molecular function as binding (GO). Both Htr5b and Gabra2 participated in neurotransmitter-related functions (GO) and neuroactive ligand–receptor interactions (KEGG). Compared to the C57BL/6 group, qRT–PCR showed that the expression levels of these genes (Hsp1a, Htr5b and Gabra2) in the DBA/1 group were significantly higher (Fig. 6c), while Gfra1 expression was significantly lower in DBA/1. The results of qRT-PCR were consistent with that in microarray analysis.

## Discussion

DBA/1 mice were more susceptible to AGSz and S-IRA than C57BL/6 mice. Previous studies have explored the influence of genetic background [7] but the differences were not completely explained. The gene expression profiles of these mice were not fully understood. In the present study, using microarray analysis, we investigated the potential roles of mRNAs and lncRNAs in the different AGSz and S-IRA susceptibilities of these two strains and identified 897 lncRNAs and 438 mRNAs that were dysregulated in the brainstems of DBA/1 and C57BL/6 mice.

GO and KEGG analyses revealed that the significantly differentially expressed mRNAs were involved in many biological functions. Comparing DBA/1 mice with C57BL/6 mice, the most highly enriched biological process in GO analysis was transforming growth factor beta (TGF-β) receptor signaling pathway for downregulated genes. In this process, the cluster of genes included *TDGF1, RASL11B, SNX6, HTRA3, ADAM9, BMPR1B, HPGD,* and *ARRB.* The TGF-β signaling pathway is involved in the dysfunction of neuronal, glial cell and blood–brain barrier (BBB) via alteration of ion channels, adenosine, glutamate, and GABA receptors [20]. Thus, disruption to the TGF-β signaling pathway can lead to changes in neuronal excitability and increase the risk of seizures [21, 22]. TGF-β signaling is also related to inflammation, which plays an important role in epileptogenesis [23]. In this study, the enrichment of downregulated genes in this term seems to contradict the epileptogenesis and thus higher susceptibility to AGSz. However, we found one study reporting that the C57BL/6 strain was resistant to TGF-β- or IL-6-induced seizures [24]. This previous finding hints to the fact that seizure in DBA/1 mice is induced by sound and is not spontaneous, and therefore, we hypothesized that lower expression of TGF-β-related genes in DBA/1 mice might represent instability due to external influences on this biological process, especially sound priming, making these mice vulnerable to AGSz. Further study is still needed.

Another important BP enriched with downregulated genes was metabolic process, with 145 genes differentially expressed. An early study demonstrated that metabolic dysfunction is evident in and may even directly cause epilepsy [25]. More specifically, one study reported that in C57BL/6 mice, a soy protein-containing diet was associated with higher susceptibility to AGSz [7]. Similarly, two recent publications indicated that a high tryptophan or ketogenic diet reduced the risk for S-IRA [26, 27]. Although one of these studies attributed these changes to gut microbes [26], combined with our data, recent evidence strongly suggests that metabolic process differences between these two strains may play an important role in terms of susceptibility to AGSz and S-IRA.

Serine-type endopeptidase inhibitor activity and hydrolase activity showed the highest enrichment score (the former due to upregulated genes and the latter due to downregulated genes) in molecular function in the GO analysis. However, the relation of these results to AGSz or S-IRA is largely unknown.

The most highly enriched cellular component terms were major histocompatibility complex (MHC) class protein complexes, enriched with upregulated genes. MHC molecules participate in negatively regulation of synaptic plasticity [28–31], and overexpression of MHC complex I protein can lead to a decreased ability to form synapses, which has been linked to several central nervous system (CNS) disorders, including autism spectrum disorders (ASDs) and schizophrenia [32, 33]. MHC may exert its effects by interacting with inflammatory cytokines that are involved in epilepsy [33, 34]. These findings suggest that MHC has the potential to be involved in AGSz and S-IRA in DBA/1 mice, although no direct evidence is currently available to support this possibility.

KEGG pathway analysis revealed that Type I diabetes mellitus (T1DM) (upregulated genes) was one of the most differentially expressed pathways. Energy metabolism through glycolysis is related to hereditary susceptibility to epileptic seizures. A previous study reported that maintenance of low blood glucose levels exerted seizure-protecting effects [35]. On the other hand, another study noted that the common mechanism in seizures and T1DM is autoimmune dysfunction [36]. In our study, other immune-mediated pathways were differentially expressed between DBA/1 and C57BL/6 mice, including antigen processing and presentation, allograft rejection and graft-versus-host disease. These findings, combined with the findings from the GO analysis (i.e., TGF-β receptor signaling pathway, MHC, etc.) implied that a broad spectrum of immune pathways might play a role in the susceptibility to AGSz in DBA/1 mice.

When considering the results of GO and KEGG together, we found that the following transcripts required extensive investigation: Htr5b, Gabra2, and Hspa1b.

The Htr5b mRNA level in DBA/1 mice was higher than that in the C57BL/6 mice, with a fold change = 6.76 (*P* < 0.05). As mentioned, serotonergic neurons were the focus of a recent study on the mechanism of S-IRA and SUDEP. Intervention to activate 5-HT neurons has been proven to reduce S-IRA in DBA/1 mice [8–10]. An electrophysiological study revealed that serotonergic neuron function was suppressed during and after seizures [37]. Since the serotonin system in the brainstem plays a key role in the regulation of breathing and arousal [38, 39], our finding on Htr5b may be related to AGSz-induced S-IRA.

We found that Gabra2 mRNA was more highly expressed in DBA/1 mice than in C57BL/6 mice (fold change = 4.15, P < 0.05). Other studies performed on DBA/2 mice have found that gamma-aminobutyric acid (GABA) and its receptors were related to AGSz and S-IRA. Elevated brain GABA concentrations protected against AGSz [40]. However, DBA/2 mice showed lower K + -induced GABA release on PND 30, which may have been related to susceptibility to AGSz [41]. Thus, we assumed that similar to DBA/2, the increased expression of the GABA receptor might be compensate for the lower GABA level in the brainstem of the DBA/1 mice compared to that in the C57BL/6 mice at certain time point. However, this hypothesis requires further examination. A previous study pointed out that the sequence and expression of the 2 subunits (gamma-1 and alpha-4) of the GABA receptor in DBA/2 mice did not differ from those in the C57BL/6 strain [42, 43]. However, these two studies were performed on the cortex and cerebellum, and the expression of the GABA receptor in DBA/1 mice had not been previously tested. Our findings suggest that, similar to those in the DBA/2 mice, the GABA receptors in the DBA/1 strain may have potentially influenced AGSz and S-IRA.

Hspa1b, which translates into heat shock protein 70 (HSP70), was expressed at significantly higher levels in the DBA/1 group than in the C57BL/6 group (fold change = 6.04, *P* < 0.05). HSP70 is an indicator of the stress response, which can be induced by various stimuli, including ischemia, traumatic injury and seizure [44–47]. A prolonged stress response is related to cell injury in neurological disease and may lead to damage to the brainstem [48]. Hence, the elevated Hspa1b expression level in DBA/1 mice may represent a high stress state in the DBA/1 strain and may be related to S-IRA susceptibility. A recent proteomic and RNA-seq study on human SUDEP cases indicated increased HSP70-positive neurons in the hippocampus, which was considered to be related to antemortem neuronal injury, such as seizures prior to death [49]. However, in our study, we found higher expression of Hspa1b in DBA/1 mice without seizures than in normal C57BL/6 mice. These results suggest that HSP70, may be more than a biomarker. It may be involved in the mechanism of AGSz, S-IRA and even SUDEP. Studies on stress are potential new prospects for mechanistic research on S-IRA and SUDEP.

In the Leitner et al. study on high-risk SUDEP patients, Gfra1 was identified as the most downregulated mRNA in the hippocampus compared to that in low-risk patients [49]. Similarly, Gfra1 was also expressed at low levels in DBA/1 mice compared to C57BL/6 mice in our study (DBA/1 vs. C57BL/6, fold change = 1.81, downregulated). Gfra1 binds to glial cell-derived neurotrophic factor (GDNF), influencing the survival and differentiation of GABAergic interneurons [50]. Decreased Gfra1 also affects the release of GDNF, resulting in more seizure activities and thus a higher risk for SUDEP [49, 51]. Combined with our findings on Gabra2, these previous findings point to the GABA system as an important modulator for both S-IRA and SUDEP.

Among lncRNAs, nuclear paraspeckle assembly transcript 1 (Neat1) is highly conserved in mammals and participates in various developmental and pathological processes. In our study, we found that the expression of Neat1 in the brainstem of DBA/1 mice was significantly lower than that in the brainstem of C57BL/6 mice (fold change = 7.51, *P* < 0.001). It has been reported that seizures can lead to a transient downregulation of Neat1, providing a scaffolding function for regulating ion channels and thus was believed to act as a protective mechanism for restoring neuron functionality after seizure [52]. We presume that Neat1 had the ability to participate in forming an electrical barrier against spreading depolarization, which has been found to be closely related to S-IRA and SUDEP [37, 53], by increasing the threshold for propagating. The relatively low Neat1 level in the brainstem of DBA/1 mice represents a deficiency in response to seizures, making these mice vulnerable to postictal electroencephalogram suppression and leading to their high susceptibility to S-IRA.

A number of antisense lncRNAs (*n* = 9) and lincRNAs (*n* = 115) were found to be dysregulated in a coexpression analysis. Among the differentially expressed lncRNAs, intergenic lncRNAs were the most common, while exon sense-overlapping lncRNAs ranked second. Among these lncRNAs, Gm14201 (ENSMUST00000117627) was the most markedly upregulated lncRNA, with a fold change = 35.75, and AK005187 (uc009bwo.2) was the most notably downregulated lncRNA, with a fold change of 326.12. The functions of these lncRNAs remain unclear. However, with such marked fold changes, these lncRNAs may have critical functions in the regulation of development and may be potential biomarkers for certain physiological processes.

In our coexpression analysis, 2010012P19Rik was the most differentially expressed antisense lncRNA and was associated with the significantly downregulated adjacent coding gene *Tnfsf12-Tnfsf13. Tnfsf12-Tnfsf13* is also known as the *TWE-PRIL* gene. The gene is, in fact, a hybrid transcript of *TWEAK* and *APRIL.* One study reported that knocking down *TWE-PRIL* enhanced axonal growth of sympathetic neurons [54]. Although sympathetic neuron function in AGSz and S-IRA has not yet been studied in DBA/1 mice, a clinical case reported that SUDEP patients could present with sympathetic hyperactivity [55]. Thus, it is reasonable to deduce that downregulation of *Tnfsf12-Tnfsf13* enhanced sympathetic neuron function by stimulating axon growth, leading to hyperactivity related to S-IRA.

A number of differentially expressed lincRNAs were detected. Our analysis showed that a majority of lincRNAs and their nearby coding genes shared the same direction of expression change. The most differentially expressed lincRNA was AA388235, with the upregulated downstream mRNA H2-Dma and the downregulated upstream mRNA B3galt4. The determination of the biological functions and detailed regulatory mechanisms of these lincRNAs requires further exploration.

## Limitation

There are several limitations to this research. First, the sample lacked anatomical precision. In this study, the whole brainstem was used to extract RNA, leading to high heterogeneity in cell types and nuclei. Thus, further separation of tissue via anatomical methods or single-cell sequencing of certain nuclei is recommended. Second, the DBA/1 mice were not tested for AGSz or S-IRA. This choice was based on a consideration that seizure itself, as well as S-IRA, which potentially influence the expression of RNA. Notably, the susceptibility of DBA/1 mice to S-IRA increased after priming by daily stimulation with sound [6]. Thus, testing the effect of priming on the RNA expression of DBA/1 mice may provide insight into the acquisition of susceptibility to S-IRA. Third, anesthesia was given according to ethics considerations. Even though we managed to dissect the brainstem as soon as possible after anesthesia, isoflurane inhalation may have influenced the expression of RNA. The determination of the extent to which this drug may have affected RNA requires further study. Additionally, the sample size of this study was small, as only 4 mice from each group were tested by either microarray or qRT–PCR.

## Conclusions

Our findings showed that a number of lncRNAs and mRNAs were differentially expressed between the brainstems of DBA/1 and C57BL/6 mice. We found TGF-β signaling and metabolic process may contribute to the differential sensitivity to AGSz and S-IRA. Also, many differentially expressed mRNAs such as Htr5b, Gabra2, Hspa1b and Gfra1 could potentially influence the susceptibility. Finally, current evidence suggested that lncRNA Neat1 and 2010012P19Rik might exert effect on AGSz and S-IRA. These findings provide new directions in the study of AGSz, S-IRA and even SUDEP.

## Supplementary Information

**Additional file 1.** The details of all differentially expressed lncRNAs.

**Additional file 2.** The details of all differentially expressed mRNAs.

**Additional file 3.** Supplementary Table based on fold change ≥ 2.0 and FDR<0.05.

**Additional file 4.** Supplementary Figure based on fold change ≥ 2.0 and FDR<0.05.

## Data Availability

The datasets presented in this study can be found in online repositories and have been deposited with the Gene Expression Omnibus (GEO) under the project accession number GSE152931 (https://www.ncbi.nlm.nih.gov/geo/query/acc.cgi?acc=GSE152931).

## Abbreviations

5-HT: 5-hydroxytryptamine
AGSz: Audiogenic seizures
ASD: Autism spectrum disorders
BBB: Blood-brain barrier
BP: Biological processes
CC: Cellular components
CNS: Central nervous system
FDR: False discovery rate
GABA: Gamma-aminobutyric acid
GDNF: Glial cell-derived neurotrophic factor
GO: Gene ontology
HSP70: Heat shock protein 70
KEGG: Kyoto Encyclopedia of Genes and Genomes
lincRNA: Long noncoding intergenic RNA
lncRNAs: Long non-coding RNAs
MF: Molecular function
MHC: Major histocompatibility complex
NEAT1: Nuclear Paraspeckle Assembly Transcript 1
PND: Postnatal day
qRT-PCR: Quantitative Real-time polymerase chain reaction
S-IRA: Seizure-induced respiratory arrest
SPF: Specefic pathogen free
SSRI: Selective Serotonin Reuptake Inhibitor
SUDEP: Sudden Unexpected Death in Epilepsy
T1DM: Type I diabetes mellitus
TGF-β: Transforming growth factor beta
