# Supplementary material for: Gene expression profile for different susceptibilities to sound stimulation: a comparative study on brainstems between two inbred laboratory mouse strains
Source: BMC Genomics. 2022 Nov 30;23:783. doi: 10.1186/s12864-022-09016-3 (PMC9710100; doi:10.1186/s12864-022-09016-3)
Supplement: Supplementary file 3 — Additional file 3. Supplementary Table based on fold change ≥ 2.0 and FDR<0.05. [file 12864_2022_9016_MOESM3_ESM.docx]

**Supplementary Table:** sensitivity test based on fold change ≥ 2.0 and FDR<0.05.

| **Up-regulated** **lncRNAs** | | |  |  | **Down-regulated lncRNAs** | | |  |
| --- | --- | --- | --- | --- | --- | --- | --- | --- |
| **Seqname** | **GeneSymbol** | **Fold Change^*^** | **FDR** |  | **Seqname** | **GeneSymbol** | **Fold Change^*^** | **FDR** |
| ENSMUST00000117627 | Gm14201 | 35.75 | 0.0002 |  | uc009bwo.2 | AK005187 | 326.12 | 0.0000 |
| uc007uzp.1 | AK038711 | 23.50 | 0.0005 |  | uc008pwq.2 | 1500004A13Rik | 73.85 | 0.0000 |
| NR_045099 | Gm11762 | 20.65 | 0.0001 |  | NR_015498 | 1500004A13Rik | 72.49 | 0.0000 |
| ENSMUST00000173149 | H2-Bl | 17.14 | 0.0000 |  | NR_033305 | AA388235 | 66.92 | 0.0000 |
| ENSMUST00000174778 | Gm10499 | 10.86 | 0.0013 |  | AK047380 | AK047380 | 66.64 | 0.0000 |
| uc008ryr.1 | AK019984 | 10.21 | 0.0008 |  | AK047372 | AK047372 | 41.87 | 0.0005 |
| ENSMUST00000137728 | AI847159 | 9.97 | 0.0002 |  | AK143879 | AK143879 | 41.70 | 0.0005 |
| uc008mqp.1 | AK085768 | 8.95 | 0.0021 |  | AK053631 | AK053631 | 37.72 | 0.0002 |
| ENSMUST00000161336 | Agl | 8.87 | 0.0002 |  | TCONS_00025043 | XLOC_018501 | 28.09 | 0.0000 |
| ENSMUST00000151051 | Gm14029 | 7.99 | 0.0010 |  | AK043180 | AK043180 | 27.94 | 0.0000 |
| ENSMUST00000180930 | Gm26793 | 7.63 | 0.0005 |  | NR_045175 | Smc2os | 26.11 | 0.0022 |
| ENSMUST00000142000 | Ift140 | 7.03 | 0.0003 |  | uc008pws.2 | 1500004A13Rik | 25.55 | 0.0000 |
| ENSMUST00000129337 | Gm11508 | 6.73 | 0.0013 |  | AK040275 | AK040275 | 24.70 | 0.0008 |
| ENSMUST00000174018 | Grm7 | 6.25 | 0.0003 |  | AK053990 | AK053990 | 24.55 | 0.0001 |
| ENSMUST00000176545 | AA465934 | 5.99 | 0.0009 |  | uc008ouc.1 | AK007174 | 24.15 | 0.0004 |
| AK155705 | AK155705 | 5.85 | 0.0001 |  | AK157804 | AK157804 | 23.90 | 0.0008 |
| AK017289 | AK017289 | 5.79 | 0.0099 |  | ENSMUST00000178906 | Gm10593 | 23.88 | 0.0001 |
| ENSMUST00000181014 | D330041H03Rik | 5.40 | 0.0091 |  | AK047207 | AK047207 | 22.35 | 0.0003 |
| AK136371 | AK136371 | 5.39 | 0.0005 |  | AK037460 | AK037460 | 19.6­­­­7 | 0.0000 |
| AK084340 | AK084340 | 5.37 | 0.0002 |  | AK157092 | AK157092 | 19.58 | 0.0002 |

**Table S1. Top 20 differentially expressed lncRNAs between DBA/1 and C57BL/6 mice**

Notes: lncRNAs, long non-coding RNAs; FDR, false discovery rate. *DBA/1 mice vs. C57BL/6 mice

| **Up-regulated** **mRNAs** | | |  |  | **Down-regulated mRNAs** | | |  |
| --- | --- | --- | --- | --- | --- | --- | --- | --- |
| **Seqname** | **GeneSymbol** | **Fold Change^*^** | **FDR** |  | **Seqname** | **GeneSymbol** | **Fold Change^*^** | **FDR** |
| NM_001037713 | Xaf1 | 112.48 | 0.0159 |  | NM_025617 | \| Tceanc2 \| \| --- \| | 48.72 | 0.0000 |
| NM_001163810 | Tescl | 20.19 | 0.0008 |  | NM_010500 | Ier5 | 41.87 | 0.0002 |
| NM_001142938 | AK010878 | 16.23 | 0.0001 |  | NM_001161411 | Trappc12 | 34.68 | 0.0004 |
| NM_011414 | Slpi | 12.71 | 0.0004 |  | NM_024472 | Gltpd1 | 31.90 | 0.0000 |
| NM_009247 | Serpinale | 11.51 | 0.0123 |  | NM_001039533 | Pdxdc1 | 20.65 | 0.0005 |
| NM_001083918 | Gm13139 | 10.97 | 0.0005 |  | NM_001145899 | Slc15a2 | 16. 47 | 0.0006 |
| NM_001111119 | Ccnb1ip1 | 9.03 | 0.0000 |  | NM_198619 | Zfp933 | 13.51 | 0.0005 |
| NM_001001490 | Oxgr1 | 8.66 | 0.0027 |  | NM_207533 | Dbx2 | 12.88 | 0.0004 |
| NM_053127 | Pcdhb2 | 8.51 | 0.0018 |  | NM_011562 | Tdgf1 | 11.52 | 0.0015 |
| NM_019788 | Bloc1s6 | 7.98 | 0.0003 |  | NM_032002 | Nrg4 | 10.46 | 0.0003 |
| NM_022420 | Gprc5b | 7.59 | 0.0003 |  | NM_183167 | AI987944 | 9.91 | 0.0003 |
| NM_029865 | Ocel1 | 7.51 | 0.0022 |  | NM_015800 | Crim1 | 9.62 | 0.0002 |
| NM_001103158 | Gm13242 | 7.27 | 0.0021 |  | NM_145594 | Fgl1 | 9.47 | 0.0024 |
| NM_026645 | Iqcf3 | 6.61 | 0.0044 |  | NM_001130176 | Tnnt2 | 9.25 | 0.0000 |
| NM_009244 | Serpina1b | 6.50 | 0.0132 |  | NM_025922 | Itpa | 7.34 | 0.0124 |
| NM_175296 | Mael | 6.44 | 0.0469 |  | NM_030707 | Fcrls | 7.11 | 0.0003 |
| NM_175537 | Zbtb38 | 6.38 | 0.0003 |  | NM_011723 | Xdh | 6.85 | 0.0292 |
| NM_153568 | Lrrc66 | 5.97 | 0.0014 |  | NM_001033374 | Gm694 | 6.30 | 0.0249 |
| NM_010478 | Hspa1b | 5.92 | 0.0016 |  | NM_201357 | Tssc1 | 6.02 | 0.0166 |
| NM_001127188 | Zfp534 | 4.99 | 0.0026 |  | NM_019420 | B3galt4 | 5.80 | 0.0339 |

**Table S2. Top 20 differentially expressed mRNAs between DBA/1 and C57BL/6 mice**

Notes: FDR, false discovery rate. *DBA/1 mice vs. C57BL/6 mice

| **lncRNA types** | **KEGG pathways** | **Associated genes** |
| --- | --- | --- |
| Up-regulated lncRNAs | 1. Nicotine addiction | *GABRA2,GRIN3A* |
|  | 2. Neuroactive ligand-receptor interaction | *CORT,GABRA2,GRIN3A,PTGFR* |
|  | 3. Complement and coagulation cascades | *SERPINA1B,SERPINA1E* |
|  | 4. NF-kappa B signaling pathway | *LTA,ZAP70* |
| Down-regulated lncRNAs | 1. Glycosphingolipid biosynthesis | *B3GALT4,B4GALNT1* |
|  | 2. Endocrine and other factor-regulated calcium reabsorption | *CLTA,KLK1B3,KLK1B9* |
|  | 3. MAPK signaling pathway | *ARRB2,MAP3K12,MAP3K5,PPP3R2,SOS1,STMN1* |
|  | 4. Renin-angiotensin system | *KLK1B3,KLK1B9* |
|  | 5. Ribosome | *MRPL15,RPL29,RPLP0,RPS6* |
|  | 6. Oocyte meiosis | *PPP3R2,PTTG1,REC8* |

**Table S3. Pathways identified from comparison between DBA/1 mice and C57BL/6 mice**

| **Seqname of lncRNA** | **Gene symbol** | **Fold change* (lncRNAs)** | **Regulation of lncRNA** | **Genome relationship** | **Nearby gene seqname** | **Nearby gene**  **symbol** | **Fold change***  **(mRNAs)** | **Regulation**  **of mRNA** |
| --- | --- | --- | --- | --- | --- | --- | --- | --- |
| ENSMUST00000145435 | 2010012P19Rik | 6.0917526 | down | natural antisense | NM_001034097 | *Tnfsf12-Tnfsf13* | 2.0415635 | down |
| AK149710 | AK149710 | 3.163844 | down | natural antisense | NM_008850 | *Pitpna* | 2.4984672 | down |
| ENSMUST00000148180 | Gm15396 | 2.5550359 | down | natural antisense | NM_008437 | *Napsa* | 4.8828493 | down |

**Table S4. Differentially expressed antisense lncRNAs and nearby coding gene**

Notes: lncRNAs, long non-coding RNAs. *DBA/1 mice vs. C57BL/6 mice

| **Seqname of lncRNA** | **Gene symbol** | **Fold change* (lncRNAs)** | **Regulation of lncRNA** | **Genome relationship** | **Nearby gene seqname** | **Nearby gene**  **symbol** | **Fold change***  **(mRNAs)** | **Regulation**  **of mRNA** |
| --- | --- | --- | --- | --- | --- | --- | --- | --- |
| NR_033305 | AA388235 | 66.9199947 | down | upstream | NM_019420 | *B3galt4* | 5.7973092 | down |
| AK047372 | AK047372 | 41.8748727 | down | downstream | NM_029865 | *Ocel1* | 7.5104853 | up |
| AK143879 | AK143879 | 41.7014068 | down | upstream | NM_001193667 | *Gm1987* | 2.8931939 | down |
| AK143879 | AK143879 | 41.7014068 | down | upstream | NM_001277167 | *Gm12429* | 5.3971264 | down |
| AK053990 | AK053990 | 24.5521064 | down | upstream | NM_030707 | *Fcrls* | 7.1055169 | down |
| ENSMUST00000178906 | Gm10593 | 23.884969 | down | downstream | NM_001193667 | *Gm1987* | 2.8931939 | down |
| ENSMUST00000178906 | Gm10593 | 23.884969 | down | downstream | NM_001277167 | *Gm12429* | 5.3971264 | down |
| uc007uzp.1 | AK038711 | 23.496569 | up | downstream | NM_001001490 | *Oxgr1* | 8.6586855 | up |
| uc029usn.1 | Gm5859 | 19.0222774 | down | upstream | NM_001085530 | *Gm13298* | 3.895603 | down |
| AK037363 | AK037363 | 15.2204905 | down | downstream | NM_008911 | *Ppox* | 3.5903657 | down |
| uc029urz.1 | DQ551946 | 12.8034244 | down | downstream | NM_001085530 | *Gm13298* | 3.895603 | down |
| AK141495 | AK141495 | 12.6885049 | down | downstream | NM_001193667 | *Gm1987* | 2.8931939 | down |
| AK141495 | AK141495 | 12.6885049 | down | downstream | NM_001277167 | *Gm12429* | 5.3971264 | down |
| ENSMUST00000151374 | Snhg3 | 12.1370958 | down | downstream | NM_001081651 | *Rab42* | 3.2691229 | down |
| ENSMUST00000151374 | Snhg3 | 12.1370958 | down | upstream | NM_026039 | *Med18* | 2.3377675 | down |
| ENSMUST00000121728 | Gm13301 | 11.4196096 | down | upstream | NM_001085530 | *Gm13298* | 3.895603 | down |
| ENSMUST00000178043 | Gm3892 | 11.2523847 | down | upstream | NM_001085530 | *Gm13298* | 3.895603 | down |
| ENSMUST00000107991 | Gm3892 | 10.8301037 | down | upstream | NM_001085530 | *Gm13298* | 3.895603 | down |
| AK052053 | AK052053 | 10.6618917 | down | downstream | NM_001193667 | *Gm1987* | 2.8931939 | down |
| AK052053 | AK052053 | 10.6618917 | down | downstream | NM_001277167 | *Gm12429* | 5.3971264 | down |
| AK044013 | AK044013 | 10.5015258 | down | downstream | NM_001193667 | *Gm1987* | 2.8931939 | down |
| AK044013 | AK044013 | 10.5015258 | down | downstream | NM_001277167 | *Gm12429* | 5.3971264 | down |
| TCONS_00022718 | XLOC_016787 | 9.2558795 | down | upstream | NM_026601 | *Hyi* | 2.3865872 | up |
| NR_077063  NR_077063  AK045717  AK045717  ENSMUST000g00169242 | 2310002F09Rik  2310002F09Rik  AK045717  AK045717  Gm17167 | 8.0799981  8.0799981  7.5876419  7.5876419  7.1664076 | down  down  down  down  down | downstream  upstream  downstream  downstream  upstream | NM_001008549  NM_010116  NM_001161411  NM_201357  NM_001085530 | *Zfp658*  *Klk1b9*  *Trappc12*  *Tssc1*  *Gm13298* | 3.0944069  4.560532  34.6760869  6.0259669  3.895603 | up  down  down  down  down |

**Table S5. Top 20 differentially expressed lincRNAs and adjacent mRNAs**

Notes: lincRNAs, long intergenic noncoding RNAs. *DBA/1 mice vs. C57BL/6 mice
