# Supplementary material for: Gene expression profile for different susceptibilities to sound stimulation: a comparative study on brainstems between two inbred laboratory mouse strains
Source: BMC Genomics. 2022 Nov 30;23:783. doi: 10.1186/s12864-022-09016-3 (PMC9710100; doi:10.1186/s12864-022-09016-3)
Supplement: Supplementary file 4 — Additional file 4. Supplementary Figure based on fold change ≥ 2.0 and FDR<0.05. [file 12864_2022_9016_MOESM4_ESM.docx]

**Supplementary Figure:** sensitivity test based on fold change ≥ 2.0 and FDR<0.05.


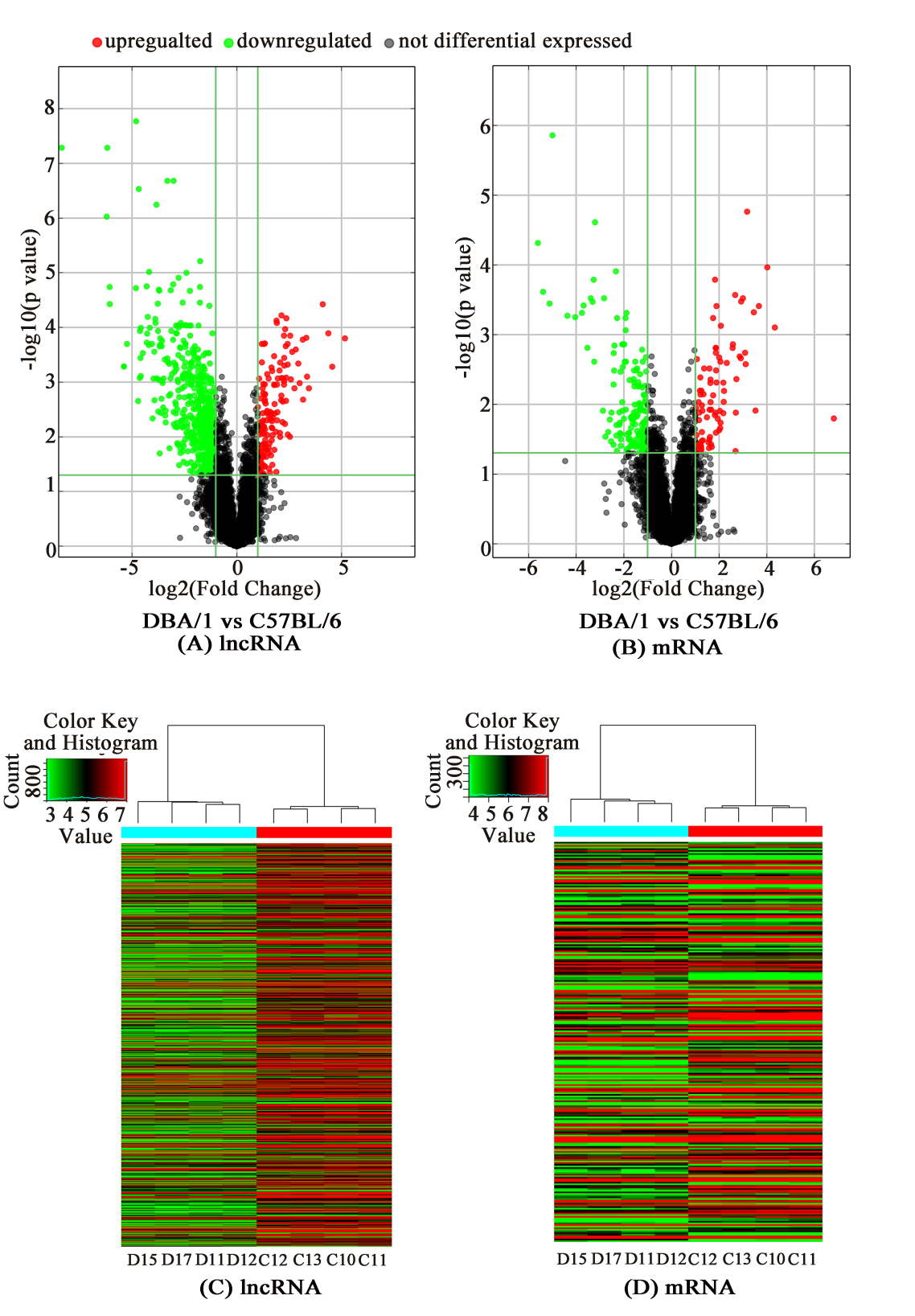


**Figure S1.** Differentially expressed lncRNAs and mRNAs in DBA/1 mice compared with C57BL/6 mice. The Volcano Plots of lncRNA (A) and mRNA (B) expression; Hierarchical clustering of differentially expressed lncRNA (C) and mRNA (D). ‘red’ indicates high relative expression, and ‘green’ indicates low relative expression. ‘C’ and ‘D’ respectively represent C57BL/6 and DBA/1 group (each group with four mice). A total of 680 lncRNAs and 246 mRNAs were differentially expressed between the brainstem of DBA/1 and C57BL/6 mice (fold change ≥2, FDR<0.05), including 145 up-regulated, 535 down-regulated lncRNAs, and 85 up-regulated, 161 down-regulated mRNAs.


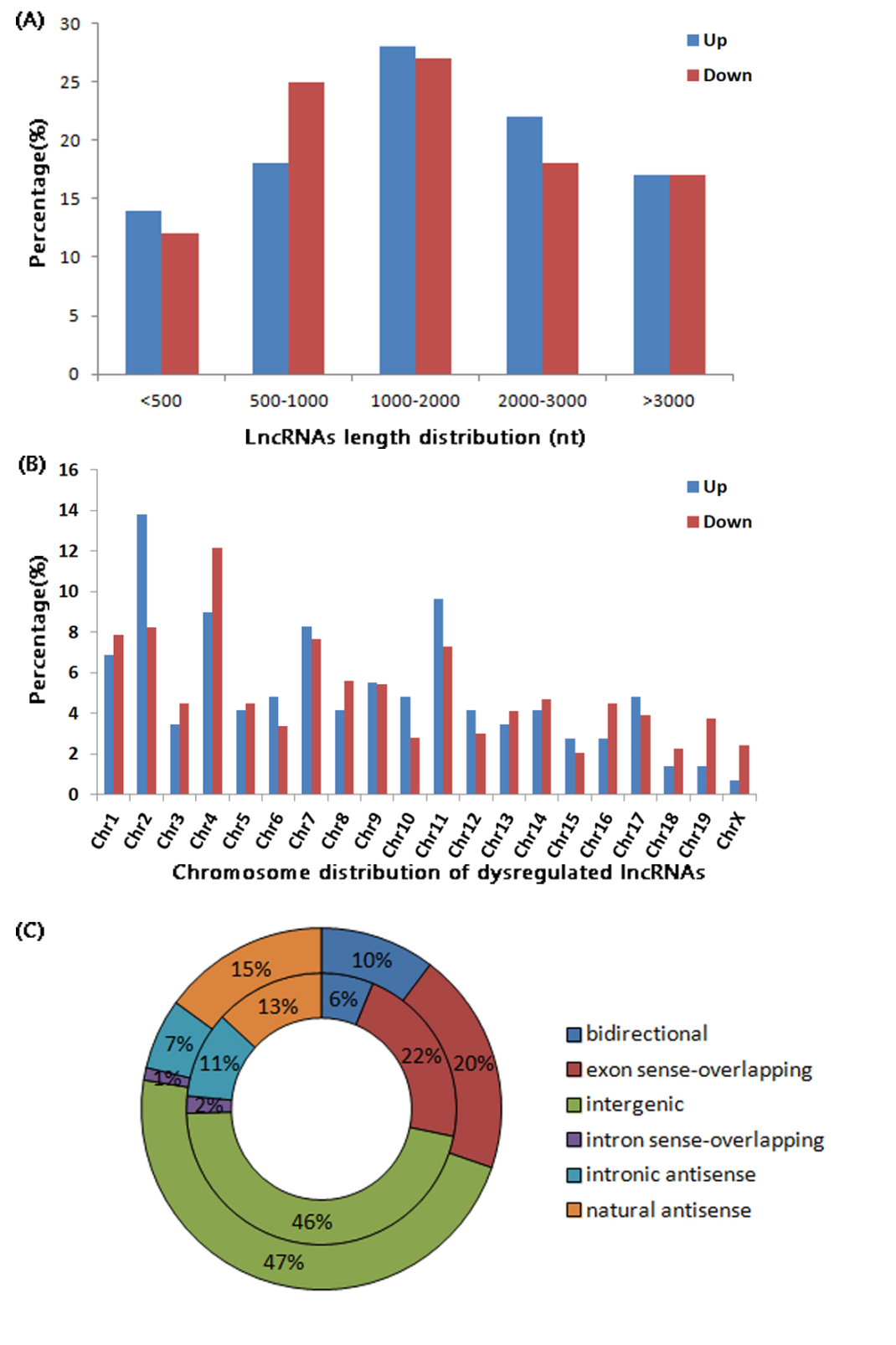


**Figure S2.** (A) The percentage of the length distribution of differentially expressed lncRNAs; (B) The percentage of the chromosome distribution of differentially expressed lncRNAs; (C) The types of differentially expressed lncRNAs. Up-regulated lncRNAs were showed in the inner circle, and down-regulated lncRNAs were described in the outer circle.


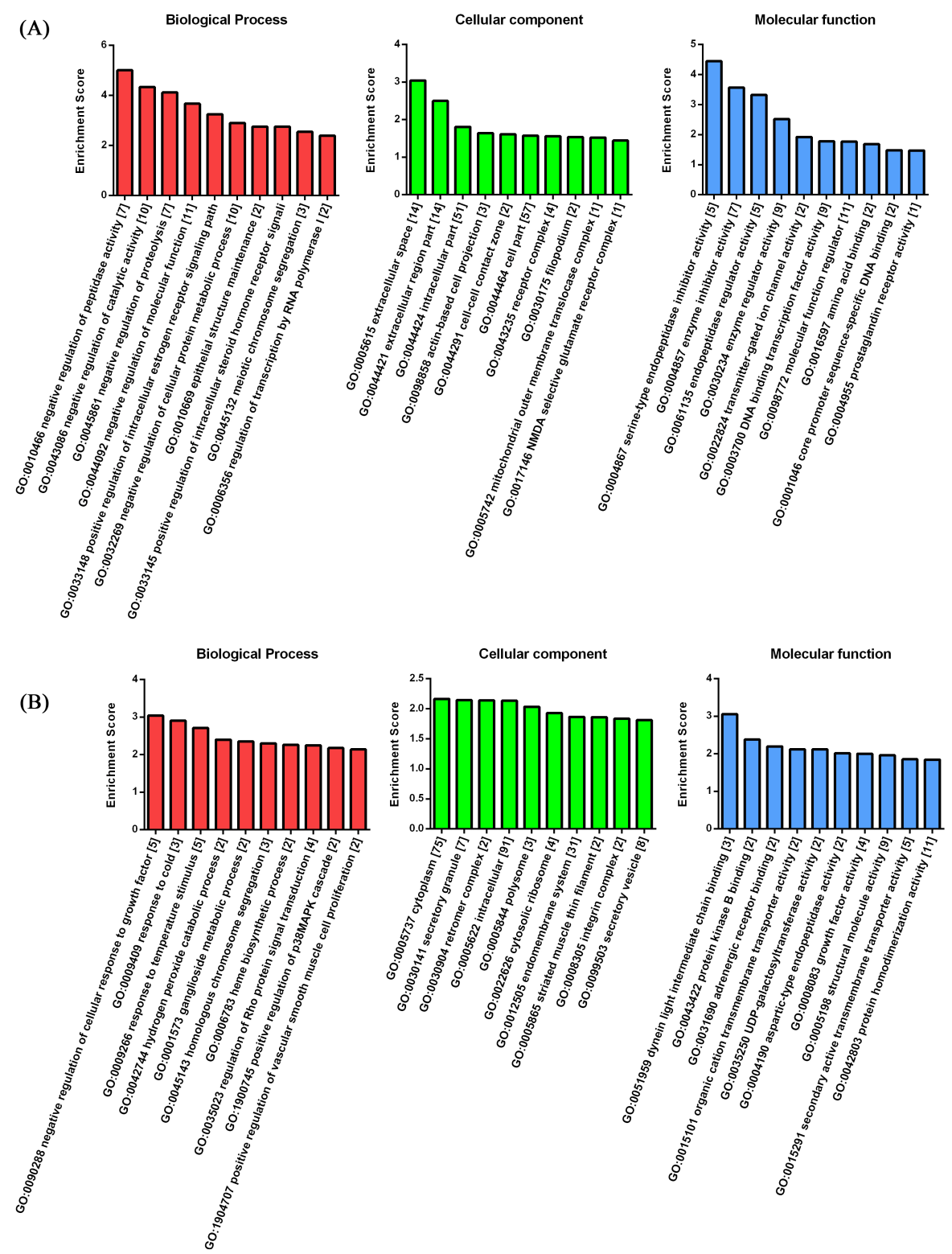


**Figure S3­­.** GO analysis comparing DBA/1 group with C57BL/6 group. (A) Top 10 enriched GO terms from upregulated mRNAs in biological process, cellular component, and molecular function. (B) Top 10 enriched GO terms from downregulated mRNAs in biological process, cellular component, and molecular function.


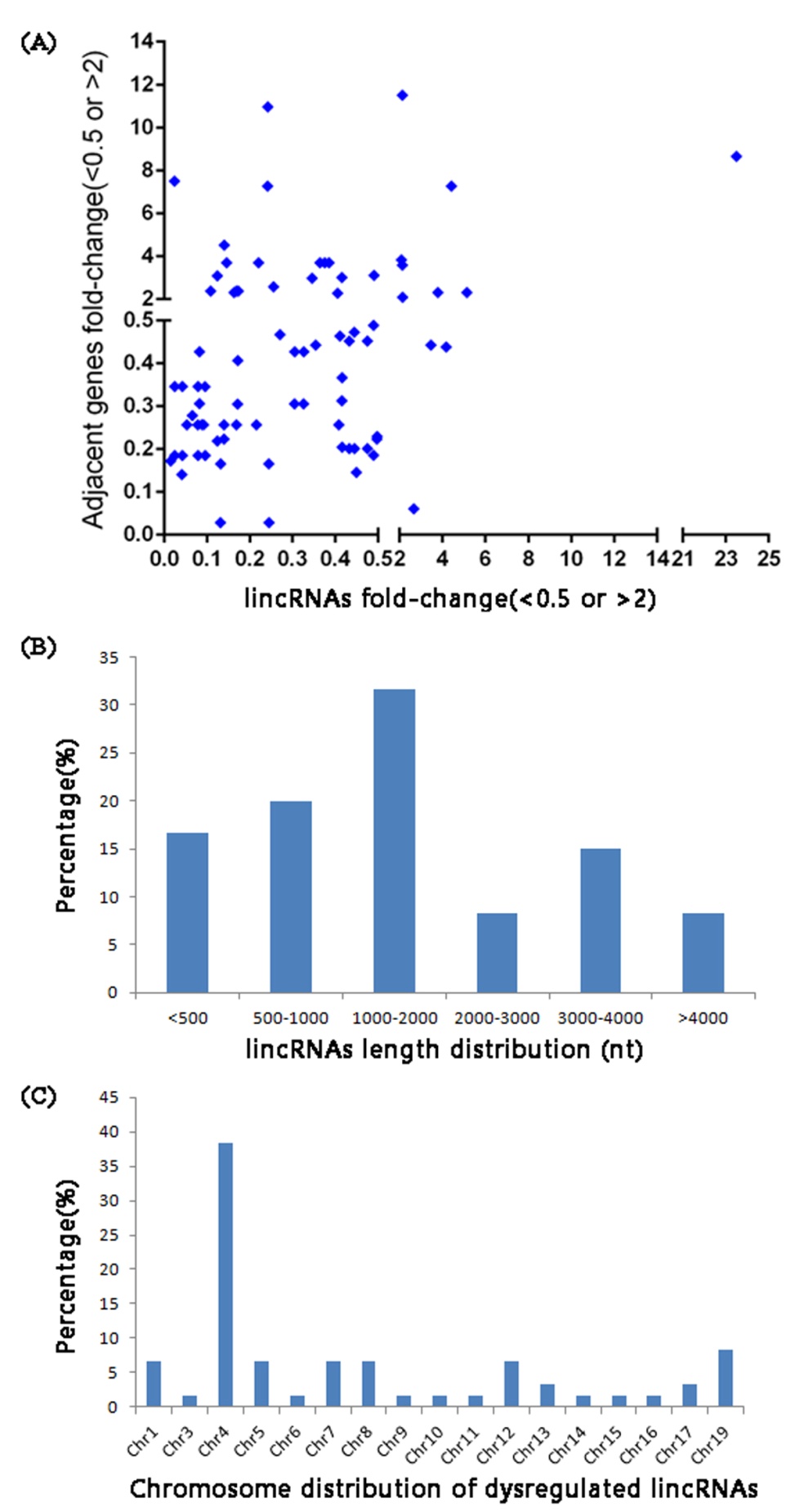


**Figure S4.** (A) Fold change of significantly dysregulated lincRNA and their differentially expressed adjacent mRNAs for DBA/1mice vs. C57BL/6 mice; (B) The percentage of the length distribution of differentially expressed lincRNAs; (C) The percentage of the chromosome distribution of differentially expressed lincRNAs.
